# Impacts of Microgravity Analogs to Spaceflight on Cerebral Autoregulation

**DOI:** 10.3389/fphys.2020.00778

**Published:** 2020-07-03

**Authors:** Marc Kermorgant, Nathalie Nasr, Marek Czosnyka, Dina N. Arvanitis, Ophélie Hélissen, Jean-Michel Senard, Anne Pavy-Le Traon

**Affiliations:** ^1^INSERM UMR 1048, Institute of Cardiovascular and Metabolic Diseases (I2MC), Toulouse, France; ^2^Department of Neurology, Institute for Neurosciences, Toulouse University Hospital, Toulouse, France; ^3^Brain Physics Laboratory, Division of Neurosurgery, Department of Clinical Neurosciences, Cambridge University Hospital, Cambridge, United Kingdom; ^4^Institute of Electronic Systems, Warsaw University of Technology, Warsaw, Poland; ^5^Department of Clinical Pharmacology, Toulouse University Hospital, Toulouse, France

**Keywords:** cerebral autoregulation, head-down bed rest, dry immersion, spaceflight analog, microgravity

## Abstract

It is well known that exposure to microgravity in astronauts leads to a plethora physiological responses such as headward fluid shift, body unloading, and cardiovascular deconditioning. When astronauts return to Earth, some encounter problems related to orthostatic intolerance. An impaired cerebral autoregulation (CA), which could be compromised by the effects of microgravity, has been proposed as one of the mechanisms responsible for orthostatic intolerance. CA is a homeostatic mechanism that maintains cerebral blood flow for any variations in cerebral perfusion pressure by adapting the vascular tone and cerebral vessel diameter. The ground-based models of microgravity are useful tools for determining the gravitational impact of spaceflight on human body. The head-down tilt bed rest (HDTBR), where the subject remains in supine position at −6 degrees for periods ranging from few days to several weeks is the most commonly used ground-based model of microgravity for cardiovascular deconditioning. head-down bed rest (HDBR) is able to replicate cephalic fluid shift, immobilization, confinement, and inactivity. Dry immersion (DI) model is another approach where the subject remains immersed in thermoneutral water covered with an elastic waterproof fabric separating the subject from the water. Regarding DI, this analog imitates absence of any supporting structure for the body, centralization of body fluids, immobilization and hypokinesia observed during spaceflight. However, little is known about the impact of microgravity on CA. Here, we review the fundamental principles and the different mechanisms involved in CA. We also consider the different approaches in order to assess CA. Finally, we focus on the effects of short- and long-term spaceflight on CA and compare these findings with two specific analogs to microgravity: HDBR and DI.

## Introduction

**FIGURE 1:**
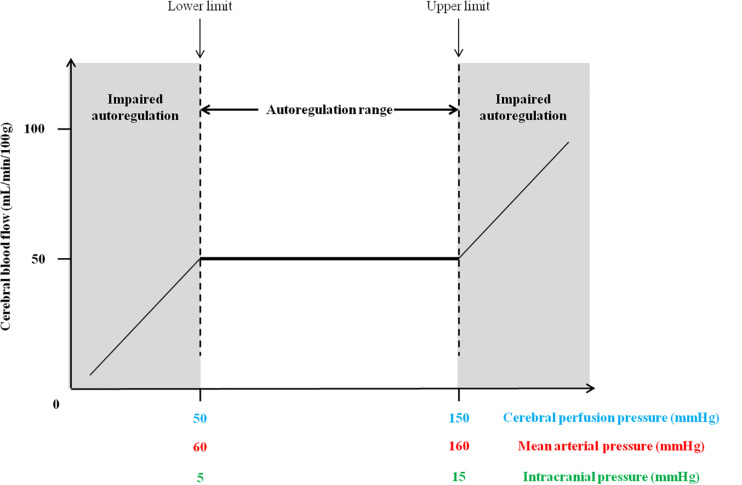
Theoretical cerebral autoregulation curve with the autoregulation plateau where cerebral blood flow remains constant with the upper and lower limits of the autoregulatory range.

Analogs to microgravity for cardiovascular deconditioning such as head-down bed rest (HDBR) and dry immersion (DI) are essentials models for determining the effects of spaceflight on astronauts’ body. Due to the cost and the limited number of space missions, these analogs are good alternatives for gravitational research (Herranz et al., 2013; Hargens and Vico, 2016). HDBR is the most commonly used ground-based model of microgravity for cardiovascular deconditioning and the subject remains in supine position at −6 degrees head-down tilt bed rest (HDTBR) for either short periods (from 1 week to 1 month), or sometimes longer periods (>1 month). HDBR mimics cephalic fluid shift, immobilization, confinement, and inactivity. It would appear that vestibular function and gravitational stimuli are also affected, however to a lesser extent compared to those observed during spaceflight (Pavy-Le Traon et al., 2007). DI model is another approach where the subject remains immersed in thermoneutral water covered with an elastic waterproof fabric separating the subject from the water. Thus, the subject is freely suspended while remaining dry. One of the main features of DI is that imitates absence of any supporting structure for the body, centralization of body fluids, immobilization and hypokinesia observed during spaceflight (Navasiolava et al., 2011a). DI impacts a wide range of physiological mechanisms such as a diminution in neuromuscular system (Grigorieva and Kozlovskaya, 1983; Kozlovskaya et al., 1984), an alteration in cardiovascular system associated with sympathoexcitation (Iwase et al., 2000), a possible impact on intracranial pressure (ICP) (Avan et al., 2013; Rukavishnikov et al., 2013; Kermorgant et al., 2017). Tomilovskaya et al. (2019) emphasized the effectiveness of DI and its ability to induce rapid physiological changes more than others ground-based models of microgravity. During human spaceflight, the absence of gravity induces headward fluid shift and leads to cardiovascular deconditioning notably characterized by orthostatic intolerance when astronauts come back on Earth (Fritsch-Yelle et al., 1994, 1996a; Buckey et al., 1996; Meck et al., 2001; Levine et al., 2002; Blaber et al., 2011; Platts et al., 2014; Lee et al., 2015; Fu et al., 2019). Several mechanisms for post-flight orthostatic intolerance were suggested. Fritsch et al. (1992) proved that after 4- to 5-day space shuttle mission, a diminution in vagal control of the sinus node was observed in 12 astronauts. These findings suggest that an impaired vagal response may be involved in post-flight orthostatic intolerance. Buckey et al. (1996) showed that after 9–14 days of spaceflight, approximately two-thirds of the astronauts presented signs of orthostatic intolerance and those with severe signs of post-flight orthostatic intolerance had disturbed total peripheral resistances. Fritsch-Yelle et al. (1996b) corroborated these previous findings. Indeed after 8–16 days of spaceflight, approximately one-third of astronauts suffered from orthostatic intolerance had lower standing peripheral vascular resistance associated with smaller increases of plasma noradrenaline and greater decreases in systolic and diastolic pressures suggesting a hypoadrenergic responsiveness. Perhonen et al. (2001) demonstrated the reduction in left ventricular cardiac mass after a spaceflight of 10 days in four astronauts that suggesting that cardiac atrophy, by altering diastolic filling, may be involved in orthostatic intolerance. Some studies proposed that hypovolemia induces orthostatic intolerance (Waters et al., 2002; Shi et al., 2004). However, Shi et al. (2004) treated seven astronauts by restoring fluid volume with fludrocortisone (a synthetic mineralocorticoid) but this treatment failed to prevent the onset of orthostatic intolerance. The signals from vestibular system, and more specifically otolith organs, contributes to the arterial pressure control at the onset of standing (Yates and Kerman, 1998; Tanaka et al., 2009, 2012, 2014, 2017; Hallgren et al., 2015, 2016; Morita et al., 2016). Some studies also proposed that impairment of cerebral autoregulation (CA) may contribute to reduced orthostatic tolerance after spaceflight or ground-based of model microgravity (Zhang et al., 1997; Novak et al., 1998; Iwasaki et al., 2007). CA is a specific homeostatic mechanism that regulates and maintains CBF constant against any changes in fluctuation in arterial blood pressure (ABP) or ICP in order to preserve cerebral function (Lassen, 1959, 1964; Johnson, 1986; van Beek et al., 2008; Armstead, 2016; Claassen et al., 2016). Lassen (1959) described the autoregulation curve with three specific phases showing a CBF plateau distinguishing the lower limit from the upper limit of autoregulation. Above these limits, CA may be lost and CBF can no longer remain steady. Figure 1 represents the theoretical autoregulation curve. In normal adults, an average CBF is 50 mL/min/100 g of brain tissue (Lassen et al., 1960; Lassen, 1985). In normotensive and healthy adults, the CBF is well maintained with cerebral perfusion pressure (CPP) between 50 and 150 mm Hg or mean arterial pressure between 60 and 160 mm Hg (Rosner and Becker, 1984; Paulson et al., 1990; Peterson et al., 2011; Armstead, 2016). However, normal value for ICP is difficult to define. Indeed in healthy conditions, this depends on age and posture (Czosnyka and Pickard, 2004). However, a normal range was defined for ICP values: 5–15 mm Hg (7.5–20 cm H_2_O) in horizontal position (Rangel-Castillo et al., 2008) and around −10 mm Hg in vertical position (Chapman et al., 1990). CPP depends on two-factors, ABP and ICP and their relationship can be established as follows: CPP = ABP−ICP. Thus, an increased ICP would lead to a diminution in CPP which may induce a cerebral vasodilatation eliciting a reduction in CBF (Rangel-Castillo et al., 2008). The response of CA occurs in a few seconds after CPP changes in order to maintain CBF in a normal range of pressure (Paulson et al., 1990; Franco Folino, 2007; Zhang and Hargens, 2018).

In this review, we first recall the mechanisms involved in CA and the different approaches allowing to assess this homeostatic mechanism. Finally, we will seek to bring together information about the impacts of spaceflight and the most commonly used microgravity analogs on CA.

## Mechanisms Involved in Cerebral Blood Flow Regulation

For decades, at least four theories are evoked to determine the mechanisms responsible for regulation of CBF: myogenic, neurogenic, metabolic and endothelial theories (Strandgaard and Paulson, 1984; Paulson et al., 1990; Peterson et al., 2011; Armstead, 2016; Rivera-Lara et al., 2017):

• The myogenic mechanism refers to the adaptation of the vascular smooth muscle to changes in transmural pressure with the corresponding vasoconstriction and vasodilatation in response to increased and reduced pressure (Ibrahim et al., 2006). Non-selective cation channels were found in vascular smooth muscle and they are strongly involved in myogenic mechanism. Indeed, a depolarization phenomenon will induce an influx of Ca^2+^ leading to vasoconstriction (Davis and Hill, 1999). The myogenic mechanism accounts for 31% of the pressure-flow relationship (Hamner and Tan, 2014).
• The neurogenic mechanism involves the innervation of the cerebral circulation with a broad panel of neurotransmitters with vasodilator and vasoconstrictor properties. Evidence shows the implication of sympathetic nervous system. Its activation shifts the upper limit of CA curve toward higher pressures in order to protect the brain against ABP increase. In parallel, it is believed that parasympathetic nervous system plays a minor role in CA. The latter, once activated, has cerebral vasodilatory effects (Hamel, 2006). Sympathetic and parasympathetic mechanisms account respectively for 20 and 11% of the pressure-flow relationship (Hamner and Tan, 2014). Perivascular nerves and astrocytes would have the ability to sense changes in CPP and adjust accordingly the sympathetic activity (Hamel, 2006; Marina et al., 2020). The astrocytes, due to their intrinsic function, may be considered as intracranial baroreceptors and play a major role in CA (Marina et al., 2020).
• The metabolic mechanism contributes to control CBF with the help of a plethora of vasoactive mediators (ions, metabolite byproducts, neurotransmitters, etc.). The vasoactive ions (K^+^, H^+^, Ca^2+^) have mainly vasodilatory properties and an elevated concentrations of these ions may lead to vasodilatation (Faraci and Sobey, 1998; Nguyen et al., 2000). The metabolic factors (lactate, CO_2_, adenosine) possess potent vasodilatory properties (Ko et al., 1990; Li and Iadecola, 1994; Attwell and Iadecola, 2002). The vasoactive neurotransmitters (dopamine, acetylcholine, gamma-amino butyric acid, vasoactive intestinal peptide) would also contribute to cerebral vasodilation (Girouard and Iadecola, 2006; Bor-Seng-Shu et al., 2012; Fantini et al., 2016).
• The endothelial mechanism, involving vasodilators (nitric oxide, carbon monoxide, prostacyclin) and vasoconstrictors (endothelin-1, thromboxane A2, angiotensin II) secreted by the endothelium in a paracrine fashion, may also play a key role in CA (Golding et al., 2002; Rivera-Lara et al., 2017; Silverman and Petersen, 2020).

## Determination of Cerebral Autoregulation: Static and Dynamic Methods

Two methods can be applied in order to assess CA: static and dynamic methods. The static method assesses the global efficiency of the CA but not takes into account the latency, while dynamic method assesses latency and then efficiency of the CA (Tiecks et al., 1995).

The static approach consists to determine CBF changes in response to a steady-state change in ABP and necessitates the administration of vasoactive drugs (Tiecks et al., 1995). An autoregulatory index can be established as the percent change in cerebrovascular resistance related to the percent change in ABP (Tiecks et al., 1995) or percent change in CPP (Priesman et al., 2005). An autoregulatory index of 100% corresponds to perfect CA and 0% corresponds to absent CA. Microvascular CBF measurement in small cerebral arterioles can provide information about the state of CA. Continuous-wave near infrared spectroscopy and diffuse correlation spectroscopy are considered as robust quantitative method in the assessment of CA (Kim et al., 2010, 2018; Caicedo et al., 2012; Durduran and Yodh, 2014; Kainerstorfer et al., 2015; Armstead, 2016). As these methods assess CA from stabilized CBF over several minutes, these methods can be encompassed in the static approach of CA.

The dynamic approach relies on the rapid changes in ABP, the CBF response to these changes, and, in particular the time to return to its baseline values (Tiecks et al., 1995; Armstead, 2016). Several methods can be applied to determine dynamic CA. The first corresponds to the slope of cerebrovascular resistance recovery determined by the CPP to CBF ratio. An abrupt slope means an enhanced CA, while a gradual moderate slope indicates an impaired CA (Tiecks et al., 1995). A rise in CPP leads to a cerebral vasoconstriction, whereas a decline in CPP leads to cerebral vasodilation (Silverman and Petersen, 2020). CA can also be assessed by transfer function analysis between mean ABP and mean CBFV signals. A cross-spectral analysis is then applied to obtain three specific frequency-dependent parameters: transfer function coherence, gain and phase. The coherence measures the degree of linearity of the relationship between mean ABP and mean CBFV. The coherence value close to 1 indicates a strong linear relationship between mean ABP and mean CBFV with high signal-to-noise ratio, while the coherence approximating with value near zero may suggest a non-linear relationship, a presence of extraneous noise or other influencing variables (Marmarelis, 1988; Giller, 1990; Liu et al., 2015). Furthermore, a threshold of coherence over 0.5 depicts that transfer function gain and phase are valid (Diehl et al., 1995; Zhang et al., 1998). The gain reflects to what extent the transmission from the ABP signal variation impacts on the CBFV signal. The phase describes the delay between sinusoidal components of ABP signal and CBFV signal and was considered as temporal relation between these signals (Zhang et al., 1998; Liu et al., 2015). The mechanism of CA has the characteristics of a high-pass filter that dampens slow fluctuations of blood pressure (Zhang et al., 1998). The most commonly ranges used to calculate the mean values of the transfer function (coherence, phase and gain) are: very low frequency (VLF: 0.02–0.07 Hz), low frequency (LF: 0.07–0.20 Hz) and high frequency (HF: 0.20–0.35 Hz) (Zhang et al., 1998, 2009). It is now accepted that elevated coherence and gain with a reduced phase shift reflect an impaired CA (Giller, 1990; Immink et al., 2004; van Beek et al., 2008; Hamner et al., 2010; Donnelly et al., 2016). Several indices of dynamic CA have also been established and derived from transcranial Doppler and correspond to correlation coefficient of blood flow velocity with CPP (Mx) or ABP (Mxa), or slow waves in ICP with ABP (PRx). An unaltered CA is determined for Mx < 0, Mxa < 0.3, or PRx < 0, while CA is considered impaired for Mx > 0, Mxa > 0.3, or PRx > 0 (Czosnyka et al., 2001; Steiner et al., 2003; Sorrentino et al., 2011).

In addition, Nasr et al. (2014) found an inverse and strong correlation between baroreflex sensitivity and CA in healthy controls. This suggests that baroreflex modulation of the autonomic drive may play an important role on CA.

## Impacts of Spaceflight on Cerebral Autoregulation

Despite the numerous studies on spaceflight cardiovascular adaptation, the impact of microgravity on CA in astronauts remains unclear. Table 1 summarizes methods used to assess CA in spaceflight.

**TABLE 1:** List of authors and the methods used to assess cerebral autoregulation in spaceflight.

**References**	**Methods**	**Subjects**	**Time duration**
Gazenko et al., 1981	CBF	6 crewmembers	185 days
Bagian and Hackett, 1991	MCA blood flow velocity	4 crewmembers	N/A
Herault et al., 2000	CFR, Q_CA_, R_CA_	6 cosmonauts	6 months
Arbeille et al., 2001	Q_CA_, R_CA_	1–9 men + 1 woman	2 days to 6 months
Iwasaki et al., 2007	MCA blood flow velocity, CBFV, TFA (VLF, LF, HF)	6 men	1–2 weeks
Kotovskaia and Fomina, 2010	CBF	26 cosmonauts	8–438 days
Blaber et al., 2011	MCAv, CR, TFA (VLF, LF)	20 men + 7 women	8–16 days
Zuj et al., 2012	CBFV, CR, PI	6 men + 1 woman	58–199 days
Marshall-Goebel et al., 2019	JVBF	9 men + 2 women	210 ± 76 days

*CBFV, cerebral blood flow velocity; CFR, cerebral to femoral flow ratio; CR, cerebrovascular resistance; HF, high frequency; JVBF, jugular venous blood flow; LF, low frequency; MCAv, middle cerebral artery velocity; PI, Gosling pulsatility index; Q_*CA*_, middle cerebral artery mean flow velocity; R_*CA*_, cerebral artery resistance index; TFA, transfer function analysis; VLF, very low frequency.*

Gazenko et al. (1981) reported that after spending several months in space, cosmonauts experienced a dramatic fall in pulse blood filling of cerebral vessels (similar to CBF pulsatility) corresponding to an improved cerebral vasoconstriction. Alternatively, during 6-months MIR spaceflights, the cerebral vascular resistance measured by resistance index was preserved in six cosmonauts (Herault et al., 2000). However, Arbeille et al. (2001) did not observe any significant changes in cerebral perfusion after short or long duration spaceflight despite a reduction in middle cerebral artery resistance index and middle cerebral artery mean flow velocity, as well as a diminution in cardiac volume, lower limb arterial resistance and an enlargement of jugular and femoral veins in cosmonauts. Bagian and Hackett (1991) did not see any significant modifications in CBFV directly measured by transcranial Doppler in crewmembers after 10 h in-flight. Iwasaki et al. (2007) also reported in six male crewmembers after a 1 and 2-week spaceflight, a reduction in gain function in the low frequency range (0.07–0.20 Hz) with no change in CBFV. These findings indicate a preserved or even better dynamic CA on landing day. Furthermore, a large hemodynamic dataset collected over 20 years in 26 cosmonauts aboard orbital stations Salyut 7 and Mir, show maintained cerebral circulation with a stabilized CBF even after long duration exposure to microgravity (Kotovskaia and Fomina, 2010). Several assumptions can be established to explain this enhancement in CA. First of all, Iwasaki et al. (2007) hypothesized that CA improvement would be due to the impact of microgravity which raises the responsiveness of cerebral vascular smooth muscle to changes of transmural pressure. Noteworthy is the cardiovascular autonomic nervous system which is known to be altered both in short- and long-duration space flight (Ertl et al., 2000; Sigaudo-Roussel et al., 2002; Eckberg et al., 2010) interacts with CA. Moreso, the baroreflex sensitivity which plays a key role in short-term blood pressure variations has been shown to be inversely correlated to CA in healthy individuals and the supposed mechanism for inverse correlation between baroreflex sensitivity and CA is an increased sympathetic tone associated with lower baroreflex sensitivity (Nasr et al., 2014). This suggests that baroreflex modulation of the autonomic drive may play a role on CA. These may indeed be one of the underlying physiological mechanisms explaining that CA was often preserved or even enhanced in spaceflight, and, could be related to changes in baroreflex sensitivity. However inter-individual variability of baroreflex sensitivity is known to be important and due to small numbers of individuals in space studies, this key interaction between impaired baroreflex sensitivity and preserved or even enhanced CA to date may have eluded identification. Pertaining to this mechanism of interaction between autonomic nervous system and CA, prior data have shown enhanced sympathetic nervous activity during prolonged exposure to microgravity (Ertl et al., 2002). This could explain preserved or better CA in most of the studies in space flight as CA is dependent on cerebral vasomotor tone which increases with afferent sympathetic drive.

A study performed in 27 astronauts, who took part in different shuttle missions lasting few days, described an impairment in CA reflected by an increase in the gain function between CBF and blood pressure (Blaber et al., 2011). This study showed that astronauts who were orthostatically tolerant presented no signs of an impaired CA, those who were orthostatically intolerant had a severe impairment in CA on landing day. These results suggest that one of the reasons leading to presyncope in astronauts is that there is a mismatch of blood pressure and CBF. Another study demonstrated in seven astronauts, which spent several days on the International Space Station, a decrease in cerebrovascular dynamic autoregulation and CO_2_ reactivity, thus characterizing an altered CA (Zuj et al., 2012). A recent work revealed that among 11 healthy crewmembers who spent a mean of 210 days in spaceflight, 6 crewmembers presented stagnant or reverse flow in the internal jugular vein (Marshall-Goebel et al., 2019), which could jeopardize cerebral circulation (Zhou et al., 2018). They suggested that cerebrovascular alterations could be due to chronic elevation in cerebral blood pressure and chronic exposure to elevated atmospheric P_CO__2_ as seen in long-duration spaceflight. Studies performed in animal models may provide an answer, though a partial one. In contrast to the hindlimb unloading in rodent, few of them were conducted during spaceflight. However, Taylor et al. (2013) demonstrated in female C57BL/6 mice that a 13-day spaceflight induced a diminished myogenic vasoconstriction and stiffness of posterior communicating arteries and an increased maximal diameter of basilar artery suggesting that cerebral perfusion could be altered. Another study showed that spaceflight induced an altered cerebral artery vasomotor in mice. Indeed, Sofronova et al. (2015) depicted in male C57BL/6N that a 30-day spaceflight attenuated vasoconstrictor and vasodilator properties in basilar artery thus likely impairing cerebral perfusion.

## Impacts of Spaceflight Analogs on Cerebral Autoregulation

Data on CA are controversial during ground-based model of microgravity. Table 2 summarizes methods used to assess CA in analogs to spaceflight.

**TABLE 2:** List of authors and the methods used to assess cerebral autoregulation in analogs to spaceflight.

**References**	**Methods**	**Subjects**	**Time duration**	**Analogs**
Guell et al., 1979	rCBF	4 men	7 days	4° HDT
Guell et al., 1982	rCBF	6 men	7 days	4° HDT
Kawai et al., 1992	CBFV	6 subjects	5 min	6° HDT
Frey et al., 1993	MCA blood flow velocity	9 men	2 days	10° HDT
Kawai et al., 1993	CBFV	8 men	1 day	6° HDT
Satake et al., 1994	CBF	Men	N/A	6° HDT
Pavy-Le Traon et al., 1995	MCAv, CR	12 men	28 days	6° HDT
Savin et al., 1995	MCAv	10 subjects	10 min	10° HDT
Zhang et al., 1997	CBFV	11 men + 1 woman	14 days	6° HDT
Arbeille et al., 1998	MCAv, CFR	8 men	4 days	6° HDT
Heckmann et al., 1999	CBFV, PI	11 men + 2 women	1 min	80° HDT
Arbeille et al., 2001	Q_CA_, R_CA_	6 to 19 men	1 h to 42 days	6° HDT
Sun et al., 2001	CBF, CR	12 men	21 days	6° HDT
Yao et al., 2001	ACAv, MCAv, PCAv, PI, RI	6 men	21 days	6° HDT
Pavy-Le Traon et al., 2002	MCAv, CR	8 women	7 days	6° HDT
Sun et al., 2002	MCAv	8 men	4 days	6° HDT
Yasumasa et al., 2002	CBFV	8 men	1 day	6° HDT
Sun et al., 2005	CBFV	12 men	21 days	6° HDT
Greaves et al., 2007	MCAv, CR, ARMA	24 women	60 days	6° HDT
Yang et al., 2011	CBFV, S/D, PI, RI	12 men	4 days	6° HDT
Geinas et al., 2012	MCAv, PCAv, CR, TFA (VLF, LF)	17 men + 4 women	>7–8 min	90°HDT
Jeong et al., 2014	TFA (VLF, LF, HF)	18 men + 3 women	18 days	6° HDT
Yang et al., 2015	CBFV	10 men and women	15 s	10°, 25°, and 55° HDT
Marshall-Goebel et al., 2016	Blood flow velocity	9 men	4.5 h	6°, 12°, and 18° HDT
Kermorgant et al., 2017	TFA (VLF, LF, HF), Mxa	12 men	3 days	DI
Ogoh et al., 2017	CBF	12 men	3 days	DI
Kermorgant et al., 2019	MCAv, TFA (VLF, LF), Mxa	12 men	21 days	6° HDT

*ACAv, anterior cerebral artery velocity; ARMA, autoregressive moving average; CBFV, cerebral blood flow velocity; CFR, cerebral to femoral flow ratio; CR, cerebrovascular resistance; DI, dry immersion; HDT, head-down tilt; HF, high frequency; LF, low frequency; MCAv, middle cerebral artery velocity; Mxa, autoregulatory index; PCAv, posterior cerebral artery velocity; PI, Gosling pulsatility index; Q_*CA*_, middle cerebral artery mean flow velocity; R_*CA*_, cerebral artery resistance index; rCBF, loco-regional cerebral blood flow; RI, Pourcelot resistance index; S/D, CBFV systolic/CBFV diastolic; TFA, transfer function analysis; VLF, very low frequency.*

Guell et al. (1979) observed in four healthy subjects who underwent 4° HDT for 7 days, a rapid decrease *in loco*-regional CBF after 2 h followed by an increase after 48 h. In a similar study, these same authors only found in six healthy subjects an elevation *in loco*-regional CBF (Guell et al., 1982). These differences may be explained by the techniques used to measure loco-regional CBF which were different between these studies (respectively measured by transcranial Doppler and Xenon-133 techniques). Furthermore, another study also showed an increase in CBFV in six volunteers who underwent exposure to a 5-min 6° HDT (Kawai et al., 1992). These same authors described an increase in CBFV during HDT and a significant decrease in CBFV after 24 h of 6° HDT in eight healthy male subjects suggesting that CBFV may have a key role in syncope in astronauts when they come back on Earth (Kawai et al., 1993). Although Frey et al. (1993) found a diminution in blood flow velocity in the middle cerebral artery in nine men after 2 days of 10° HDT, but was inversely correlated with percent changes in retinal vascular diameters, suggested that CBF was not diminished. Satake et al. (1994), with the help of image analysis using single photon emission computer tomography, demonstrated that a significant increase in CBF in men was occurred in the basal ganglia and the cerebellum at 5 min after the onset of HDT, but not in the cerebral hemisphere. Pavy-Le Traon et al. (1995) showed in 12 healthy volunteers who underwent 28 days of HDBR, no changes in middle cerebral artery velocities recorded indirectly by transcranial Doppler indicating a preserved cerebral circulation. However, subjects presenting presyncopal symptoms had a drop in middle cerebral artery velocities indicating an impaired CA. An increase in middle cerebral artery velocity has also been observed in 10 subjects at during 10° HDT, which was restored at the end of the experiment (Savin et al., 1995). Heckmann et al. (1999) did not find any significant changes in CBFV measured by transcranial Doppler sonography but an increase pulsatility index after 1-min 80° HDT in 13 healthy volunteers. In a 4-day of HDBR study, no major changes were observed in dynamic CA in eight healthy volunteers during head-up tilt (Arbeille et al., 1998). The same authors did not find any significant changes in cerebral perfusion in men in a 42-day HDT despite an increase in cerebral artery resistance index and a decrease in middle cerebral artery mean flow velocity during the early phase of HDT (4–5 days) (Arbeille et al., 2001). Another study performed in eight healthy women showed that cerebrovascular resistance was preserved during and after a 7-day HDBR suggesting an unaltered CA. In this study, five of eight women who presented orthostatic intolerance had a time to maximum decrease in cerebrovascular resistance larger than the three women who did not, suggesting that some differences in CA may be related to orthostatic intolerance (Pavy-Le Traon et al., 2002). Consistently, Yasumasa et al. (2002) found an elevated CBFV in eight men, measured by transcranial Doppler, during the early phase (first 6 h) of 6° HDT. Moreover, a 60-day HDTBR study demonstrated that among 24 healthy women, those who exhibited few changes in orthostatic tolerance presented a preserved dynamic CA, determined by autoregressive moving average (Greaves et al., 2007). Also, Geinas et al. (2012) did not observe any significant changes in CBFV in 21 healthy young adults, even after severe changes in posture (90° HDT), although ABP was elevated compared to supine position. Similarly, a 18-day HDBR study performed in 14 healthy adults depicted a decrease in transfer function gain. These findings stated an improved dynamic CA (Jeong et al., 2014). Yang et al. (2015) found in 10 subjects an elevation in CBFV after acute consecutive exposure to randomized 10°, 25°, and 55° HDT; however, an autoregulatory correction index was applied in this study and revealed a modified but an unimpaired cerebrovascular autoregulation. Moreover, a recent work in 12 healthy male subjects who underwent 21 days of HDBR showed that autoregulatory index Mxa was reduced reflecting an enhanced dynamic CA (Kermorgant et al., 2019). Following an extensive literature search, we found only few studies that measured CA after DI. Kermorgant et al. (2017) demonstrated that the effects of 3-day DI would improve CA. Indeed, a decrease in autoregulatory index Mxa associated with an increase in the cross-spectral phase shift were observed in 12 healthy male subjects. Ogoh et al. (2017) showed in the same study that DI affected both anterior and posterior cerebral vasculature, but did not provoke a heterogeneous CBF response in each cerebral artery (internal carotid, external carotid, common carotid, and vertebral arteries) measured by Doppler ultrasonography. As previously suggested for spaceflight, one suggestion for CA improvement is that microgravity could increase the responsiveness of cerebral vascular smooth muscle to changes of transmural pressure (Iwasaki et al., 2007). However, plasma volume may also play a key role in this enhancement. Indeed, previous authors found that a diminution in plasma volume provoked a reduction in transfer function gain (corresponding to an enhanced CA), while when plasma volume was restored by volume loading, transfer function gain was increased (corresponding to an impaired CA) (Ogawa et al., 2007, 2009; Jeong et al., 2014). Consistently, several studies showed a reduction in plasma volume both in HDBR (Convertino et al., 1990; Iwasaki et al., 2000; Belin de Chantemèle et al., 2004) and in DI (Navasiolava et al., 2011b; de Abreu et al., 2017).

However, although Zhang et al. (1997) did not observe any significant modifications in CBFV in 12 subjects. The authors documented an impaired CA reflected by a greater decline in CBF during lower body negative pressure after 2 weeks of HDBR. They also speculated that impairment of CA may be involved in orthostatic intolerance after bed rest. Sun et al. (2001) performed a 21-day HDT study with or without the effects of lower body negative pressure in 12 healthy male subjects in order to investigate potential changes of CBF. Six of twelve subjects were allocated in control group (without lower body negative pressure) and they exhibited an increase in cerebral vascular resistance and a decrease in CBF measured by rheoencephalogram. They further showed, a diminution in CBFV measured by transcranial Doppler sonography, on the last day of a 4-day HDT study conducted in eight healthy male volunteers. The same authors further identified a reduction in CBFV determined by transcranial Doppler technique in 12 healthy male volunteers during a 21-day 6° HDTBR study (Sun et al., 2005). Yao et al. (2001) confirmed these previous reports with a 21-day HDT study performed in six healthy male. Indeed, they described in these subjects a reduced systolic blood velocity of both side middle cerebral artery and mean blood velocity of right middle cerebral artery. Moreover, Yang et al. (2011) found changes in cerebrovascular functions in six healthy male subjects after a 4-day HDT with reductions in CBFV, pulsatility index, resistance index, and S/D (corresponding to CBFV systolic/CBFV diastolic). All these parameters returned to basal values after HDBR. Similar findings were observed with a reduction in CBFV, measured with phase-contrast magnetic resonance imaging, in nine healthy male subjects after short-term exposure (∼4.5 h) to randomized 6°, 12°, and 18° HDT angles (Marshall-Goebel et al., 2016). The mechanisms involved in this impairment remain unclear. However, alterations in CA may be explained by a potential rise in ICP. Indeed, Kermorgant et al. (2017) found that ICP (indirectly measured by optic nerve sheath diameter) was negatively correlated with the autoregulatory index (Mxa) and coherence after 3-day DI in healthy volunteers. Despite an overall improvement in CA, subjects with low values of optic nerve sheath diameter (corresponding to low values of ICP) presented a better CA than subjects with high values of optic nerve sheath diameter (corresponding to high values of ICP). Moreover, it has been shown that patients with head-injury with the presence of an elevated ICP presented an impaired CA (Czosnyka et al., 2001). However, many studies showed that pronounced HDT led to a rise in ICP. Chin et al. (2015) showed that 3 min Trendelenburg position (corresponding to ∼30° HDT) were sufficient to induce an elevation in optic nerve sheath diameter compared to supine position in anesthetized patients. Another study performed in healthy subjects showed an elevation in optic nerve sheath diameter after 5 h in 18° HDT position (Marshall-Goebel et al., 2017). Petersen et al. (2016) also demonstrated in ambulatory neurosurgical patients that ICP (measured directly by tip-transducer) was significantly increased with HDT (10°: 14.3 mm Hg and 20°: 19.0 mm Hg) compared to supine position (0°: 9.4 mm Hg). Animal models may provide further explanations. Indeed, tail-suspended rat model is an efficiency method to reproduce major physiological changes observed in space flight, just as long-term bed rest (Morey-Holton and Globus, 2002; Morey-Holton et al., 2005; Carpenter et al., 2010; Chowdury et al., 2013). Studies performed in hindlimb suspension in rat showed that this alteration can originate from several factors including, an elevation in cerebrovascular resistance accompanied by a reduction in blood flow, hypertrophy phenomenon, a rise in myogenic tone. and vasoreactivity in cerebral arteries (Geary et al., 1998; Wilkerson et al., 1999; Morey-Holton and Globus, 2002; Morey-Holton et al., 2005; Lin et al., 2009).

## Conclusion

Either in spaceflight or in ground-based model of microgravity, most of short-term studies show a preserved or even an improved CA. However, long-term studies depict an impairment in CA. One of the main reasons for the discrepancy may depend on baseline orthostatic tolerance. Indeed, it would seem that subjects who are orthostatically tolerant have a preserved or even an enhanced CA. In contrast, subjects who are orthostatically intolerant would present an impaired CA (Blaber et al., 2011). These differences can also be attributed to the methods used to assess CA and the interpretation of each measure should be made cautiously (Tzeng et al., 2012). Furthermore, as highlighted by Armstead (2016) and Zhang and Hargens (2018), none efficient methods exist to accurately measure CBFV. The assessment of CA should be compared by gender. In fact, several studies showed gender differences in CBFV (Ackerstaff et al., 1990; Marinoni et al., 1998), cerebral vasomotor (Karnik et al., 1996) and cerebrovascular reactivities (Kastrup et al., 1997), CA both in young and old adults (Wang et al., 2005; Deegan et al., 2009). Moreover, regional differences were observed between anterior and posterior cerebral circulation and this should have been taken into account during the assessment of CA (Roth et al., 2017). Petersen and Ogoh (2019) also suggested that dynamic CA could be over- or underestimated due to the fact that gravitational stress may alter regional arterial pressure and ICP/CPP differently. Additional studies are needed in order to determine more accurately the impact of microgravity on CA. Finally, although residual effects of gravity remain, HDBR and DI could be considered as robust ground-based analogs to spaceflight for studying CA in humans during microgravity.

## Author Contributions

MK, NN, MC, DA, OH, J-MS, and AP-L wrote and edited the manuscript. All authors contributed to the article and approved the submitted version.

## Conflict of Interest

The authors declare that the research was conducted in the absence of any commercial or financial relationships that could be construed as a potential conflict of interest.
